# Developing Methods for Observing Awe Narration in Psilocybin-Assisted Therapy

**DOI:** 10.3390/healthcare14111589

**Published:** 2026-06-05

**Authors:** Elise C. Tarbi, Ian Bhatia, Nabil Balach, Suzannah Buehler, Magdalena Demeo-Meres, Cailin Gramling, Tej Thambi, Julia Hart, Maija Reblin, Donna M. Rizzo, Robert Gramling, Manish Agrawal, Emily Manetta

**Affiliations:** 1 Department of Nursing, University of Vermont, Burlington, VT 05405, USA; 2 Department of Family Medicine, Vermont Conversation Lab, University of Vermont, Burlington, VT 05405, USA; 3 Graduate Program in Complex Systems and Data Science, College of Engineering and Mathematical Sciences, University of Vermont, Burlington, VT 05405, USA; 4 College of Letters and Science, University of California, Los Angeles, Los Angeles, CA 90095, USA; 5 Department of Civil & Environmental Engineering, University of Vermont, Burlington, VT 05405, USA; 6 Sunstone Therapies, Rockville, MD 20850, USA; 7 Linguistics Program, University of South Carolina, Columbia, SC 29208, USA

**Keywords:** awe, interpersonal communication, psilocybin, psychedelics, psychedelic-assisted therapy

## Abstract

**Background:** Understanding the benefits of psychedelic-assisted therapy (PAT) will require scientific attention to the causal interaction between the therapeutic context and process. Measuring what actually happens during PAT in large-scale studies will be an essential component of this work. **Objective**: We aim to develop and preliminarily evaluate the feasibility and reliability of a direct observation coding system for narrations of awe experiences during PAT, one hypothesized therapeutic mechanism. **Methods**: We analyzed 32 PAT clinical trial encounter recordings involving eight participants from a Phase 2 clinical trial study of psilocybin-assisted therapy in advanced cancer. Using a conceptually grounded structured codebook, two human coders independently identified start and stop times for moments exhibiting definitional characteristics of awe narration, including expressions of vastness, need for accommodation and ineffability. We used coder agreement and degree of confidence to refine the coding system. **Results**: During 16,760 total minutes of video, coders collectively recorded 246 moments of awe narration. Of those moments, 42% (104/246) were identified by one coder and 58% (142/246) by two coders. Coders felt substantially more confident in their judgments about a moment of awe when vastness was present compared to when vastness was absent (OR: 4.3; 95% CI: 2.4, 7.8). Iterative refinement of the coding system led to accommodation being operationalized as two distinct components: an initial cognitive disruption followed by variable engagement in the process of accommodation. **Conclusions**: Awe narration is directly observable using explicit definitional criteria. This work provides the empirical foundation for scalable coding systems of awe narration during PAT.

## 1. Introduction

Understanding the mechanisms of psychedelic-assisted therapy (PAT) is essential preparation for safe and effective implementation at the population level [1]. Identifying key causal pathways which achieve desired outcomes will require substantial scientific attention to what *actually happens* during PAT within the multilevel contexts in which therapy occurs (e.g., cultures, languages, sources of suffering).

Two system-level changes in healthcare address methodological barriers to large-scale direct observation studies necessary to inform high-quality study of PAT mechanisms. First, ambient recording of clinical encounters for the purposes of AI-generated session documentation creates a data infrastructure of massive scale [2]. Second, advances in natural language processing and machine learning methods make valid, meaningful and de-identified measurement of complex clinical interactions feasible [3,4,5,6]. One approach to developing scalable direct observation systems is to create valid human labeling methods that identify clinically important moments. This offers two complementary scientific functions: to better understand the phenomena of interest and, once refined, the “ground truth” for training computational methods that automatically identify and characterize those moments.

Here, we describe the development of a direct observation coding system for the context of PAT, the narration of awe, which will inform future PAT mechanistic work. Awe experiences are associated with improved quality of life [7,8,9,10] and may signal mystical experiences [11,12], which are hypothesized to contribute to the therapeutic effects of PAT [11,12,13].

## 2. Methods

### 2.1. Overview

This is a descriptive study of 32 PAT clinical trial encounter recordings to identify directly observable moments in which participants narrate experiences of awe. Using a conceptually grounded structured codebook, two human coders independently identified start and stop times for moments exhibiting definitional characteristics of awe narration. We used co-identification and coder degree of confidence about each moment’s overall expression of awe to refine the coding system.

### 2.2. Participants and Setting

This sample includes participants with cancer and major depression who completed a recent Phase 2, open-label study of psilocybin-assisted therapy in which all participants received psilocybin [14,15]. Data include audio and video recordings of all preparation, dosing and integration encounters for each of the 8 participants over the three-week course of PAT. We purposefully selected participant–therapist dyads to maximize gender identity combinations among therapist (n = 7) and participant (n = 8) pairs.

### 2.3. Conceptual Definition of Awe

Keltner and Haidt (2003) foundationally define awe as a complex emotion that involves an experience of perceived vastness and a need for cognitive accommodation to adjust to overwhelmed mental structures [16].

### 2.4. Candidate Features of Awe Narration

The overwhelm and adjustment inherent to awe’s impact on cognitive processing can present communicative challenges for those attempting to explain it. Therefore, our initial direct observation coding system included the following three key features of *what the speaker said and how they said it*.

(1) Vastness: We defined vastness as expressions relating to anything being much larger than the self, with physical, social, and conceptual sub-types [16,17,18]. The “self” during PAT may be experienced at any size dimension, including infinitely small; thus, coder judgments about the presence of vastness reflect the expressed comparative relationship to self [17,18].

(2) Need for Accommodation: We considered expressions of the “need for accommodation” to relate to discussions that suggested a violation to one’s normal understanding of the world that would require a shift in mental structures to make sense of this new experience [16].

(3) Ineffability: Expressions of “ineffability” could be recognized when participant descriptions suggested something was too extreme to be expressed in words [19,20]. Manetta and Bhatia [20] define ineffability in serious narratives as the temporary inability of the narrator to express themselves adequately due to the profound nature of the experience and their affective response. They identify ineffability as characterized by long pauses (+2 s), word- and sentence-level restarts, and dedicated discourse markers (i.e., ineffability *just*). This stands in sharp contrast to typical disfluencies, which are often attributable to normal utterance planning and cognitive burden and tend to go unnoticed/unmentioned by both conversational partners.

The following participant quote contains all primary features of awe, which have been bolded and [*bracketed*] for emphasis:

“It was one of the **most profound and um meaningful experiences of my life** [*vastness*]. Shocking of its revelations, daunting at times, sometimes **unbearably beautiful** [*need for accommodation*], and mystical, divine, and it opened up **a whole world of ideas and thoughts** [*vastness*]. **Any metaphor I could use would be inadequate to describe it** [*ineffability*].”

(Participant T, Integration.)

### 2.5. Analyses

**Figure 1:**
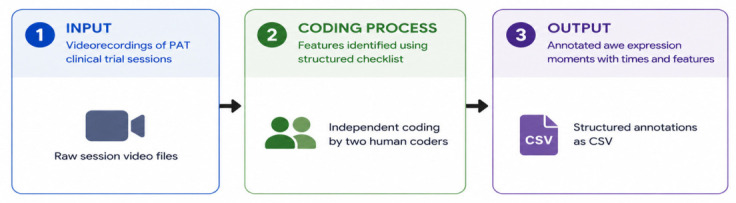
Coding workflow.

Coders independently double coded all participant–therapist encounter videos, pausing after the first four participants to evaluate coder feedback and refine operational feature definitions. Coders identified start and stop times for each instance of awe narration, defined inclusively as conversational moments containing at least one of the following: vastness, need for accommodation and ineffability. Our coding workflow is diagramed in Figure 1.

For each identified moment, coders also responded to the following question, “How confident am I that this moment meets our coder definition? (low, moderate, high)”. This gradient approach to coding, from low to high confidence, allowed us to incorporate the ambiguity of coding [21] with the complex and socially constructed concept of awe, as we have done previously in PAT settings [15].

When coders encountered a moment that “felt like an expression of awe” but exhibited none of the candidate features, we marked those separately as “boundary moments”. Consider the moment below, which exhibited many secondary features in our system (metaphor, beauty), but was not coded as an awe expression because it lacked vastness.

“It’s so many things in life. It’s the taste of your favorite ice cream. It’s the smell of your favorite flower. It’s the sight of your favorite child, your wife, your loved ones.”

(Participant T, Integration.)

Examining these events helped to ensure that our definition was sufficient for capturing awe narration as we had intended and to help with scientific transparency regarding the types of adjacent moments that would not be captured by our coding system.

Throughout our coding process, real-time notes about coders’ experience observing moments of awe were documented as an audit trail for review at regular research meetings [22]. Coder debriefing involved informal prompts about the overall coding experience, such as aspects of the coding process that were interesting or challenging. Clarifications needed to improve operational definitions and suggestions for improving the recognition of narration of awe experiences were also discussed.

The frequency and distribution of awe narration features were calculated for preparation, dosing, and integration encounters. We considered a moment of awe narration to be co-identified when any portion of the event duration observed by one coder overlapped with that of the other coder. We included situations of a common border (e.g., start time in minutes and seconds of video time identified by one coder is identical to the end time for the other coder) to count as overlap. We calculated Cohen’s kappa for inter-rater agreement and used the average duration of identified awe moments as the unit of analysis. When presenting measures of association, we use unadjusted odds ratios (ORs) and 95% confidence intervals (CIs). We used mixed-effects logistic regression modeling to evaluate potential confounding by clustering of unmeasured phenomena for the 8 participant experiences.

Institutional review boards at Advarra (parent clinical trial) and the University of Vermont (analyses of trial video data) approved all study protocols. Participants completed separate informed consent for analysis of clinical trial video recordings.

## 3. Results

The median age for participants in this sample was 59 years (range: 30–78); half were female (50%), and all identified as white (100%), with one individual also identifying as Asian American/Pacific Islander (12.5%). Cancer diagnosis varied, including breast (37.5%), kidney (25%), lymphoma (25%), and colon cancer (12.5%). Twenty-five percent had an expected survival of less than two years.

During 16,760 total minutes of video, coders collectively recorded 246 moments of awe narration with substantial inter-rater reliability (Cohen’s kappa: 0.73; 95% CI: 0.68, 0.78). Of those moments, 42% (104/246) were identified by one coder and 58% (142/246) by two coders. Of those moments coders designated with “high confidence,” 30% (31/104) were identified by one coder and 70% (73/104) by two coders. Very few awe narration events happened during preparation encounters during the first half of coding (5 of 168 total events), so we focused on dosing and integration encounters after coding the first four participants.

### 3.1. Vastness

Upon reflection, coders determined that vastness was highly salient and an obvious indicator for awe expressions. Coders felt substantially more confident in their judgments about a moment of awe when vastness was present compared to when vastness was absent (OR: 4.3; 95% CI: 2.4, 7.8). This association was not attenuated when adjusting for potential clustering by specific participant experience (OR_adj_: 4.1; 95% CI: 2.1, 8.0), nor did it differ substantially for dosing encounters and preparation–integration encounters.

### 3.2. Ineffability

Awe narration frequently included intervals in which the participant seemed to struggle to find the right words to describe their experience. More than half of all awe narrations (130/237; 55%) featured ineffability, and most of these occurred during either the dosing session or the initial integration encounter the morning after the dosing experience (75%; 97/130).

### 3.3. Need for Accommodation

Observed expressions of accommodation comprised two fundamental elements. The first involves description of an initial cognitive disruption from one’s normal understanding of the world, and the second, the status of its potential accommodation. This second element raised challenges for coders, having encountered instances in the data in which participants seemed to resist or be uninterested in meaning-making or coming to an understanding of an experience outside of their mental structures. Coders were frequently uncertain about whether expressions indicated a “need” or even a “want” for accommodation. Therefore, we included situations regardless of participant intention or desire to attempt such accommodation. One participant discusses their experience in this way:

*“Well, I don’t have to be concrete about it and say, define it. I don’t have to. I could just experience it and feel it and remember it. Um, I don’t, I don’t have to put it in words.”*

(Participant H, Integration Encounter.)

Another participant even expressed experiencing relief in not needing to continue to pursue accommodation:

*Participant: It’s still all inside of me. It’s just, like, everything that happened yesterday came from inside of me.*

*Therapist: Yeah. Yeah, okay. And so what’s your thought right now about that?*

*Participant: I think--I think I’m not going to figure it out. [laugh]*

*Therapist: Okay. Okay. And what’s that like for you, not to-to know that you’re not going to figure it out?*

*Participant: I kind of like it. It feels like a relief. You know? Like I’m not-I’m not going to find the meaning, I don’t think. Like I’m not going to find that profound thing that I’m seeking. I think it’s just going to be the process of seeking, is what it is.*

(Participant P, Integration Encounter.)

**Figure 2:**
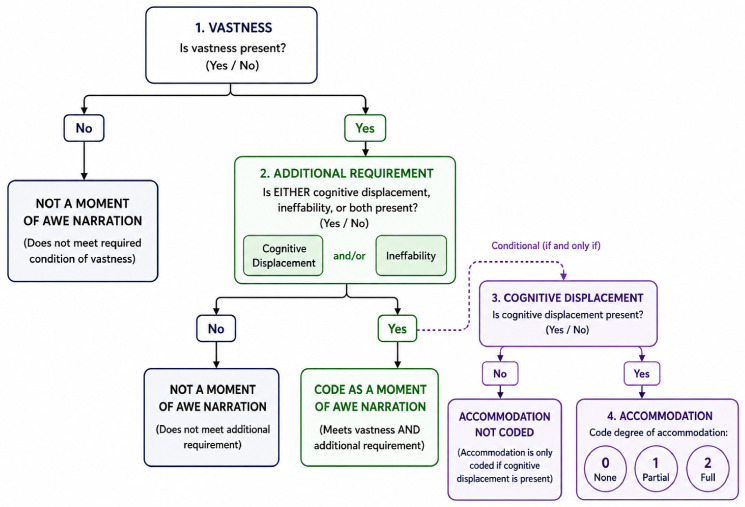
Final coding process.

Final coding definitions and illustrative examples of vastness, need for accommodation and ineffability are described in Table 1. Our final coding process is diagramed in Figure 2.

## 4. Discussion

Our findings suggest that the narration of awe experiences in psychedelic-assisted therapy is directly observable and support two empirical recommendations for coding systems. First, our findings support recommending the expression of vastness as the crucial “point of entry” for coders to confidently identify candidate awe narration moments in need of subsequent review and confirmation. Though it is possible that vastness emerged as a key feature partially due to its relative salience and ease of identification by coders, this approach aligns with Keltner and Haidt’s original proposition that vastness is foundational to the experience of awe [16], while preserving the particularity of the PAT encounter through the additional requirement of ineffability or accommodation.

Second, we suggest operationalizing the notion of accommodation by first identifying narrative evidence of an initial substantial cognitive disruption [23,24] and, if present, then proceeding to consider the potential subsequent accommodation of that disruption. This may help to disentangle the complex and challenging-to-measure construct of “need for accommodation” [25]. This nuanced characterization of the accommodation process acknowledges that some experiences of awe may never be accommodated, and that some PAT participants may be unable or uninterested in trying to do so during their therapeutic process. Conceptually, this representation of the accommodation process is aligned with newer understandings of PAT based in neuroscience, in which neural networks are first disrupted so that new neural pathways may then be mapped [26,27]. It is also in keeping with the integration model advanced in this therapeutic approach to PAT, which holds that the post-dosing sessions offer opportunities to restructure or reframe insights and integrate the experience [28].

The primary outcome of our study is the development of a direct observation coding system for use by human coders. Given the size of PAT datasets (containing thousands of hours of recorded clinical trials), our codebook may also be deployed in the future to inform automated detection of moments of awe narration. While automated machine learning can enhance the efficiency and scalability of identifying patterns in therapeutic settings, these tools—particularly those that leverage supervised learning—struggle with ambiguous or complex training data. Robust and reliable automation requires learning from a subset of accurate “training” data manually labeled by humans. Thus, human oversight and refined codebooks, such as the one developed here, are essential for quality control, validation, and ensuring the ethical considerations of the labeled data.

This study has important limitations. First, our observations arise from a small sample of PAT participants. Second, our sample lacked sufficient ethnic, racial, and linguistic representations to understand how awe narration may differ across populations and languages. It may well be that this coding system requires adaptation for deployment in a larger, more diverse sample. Third, our analyses do not attempt to quantify nor qualify the degree of awe experienced by participants (awe as an affective state) but instead investigate the nature of awe narration. We do note that in this study, awe narration often occurred simultaneous to or only shortly following the experience of awe, better positioning narration as an observable proxy for experience. Further work will be required to evaluate the validity in larger populations and the degree to which narrated awe is associated with both the internal experience of awe and the beneficial outcomes of PAT.

## 5. Conclusions

Our investigation of clinical trial PAT encounter recordings supports the feasibility of direct observation of awe narration and that coders can categorize moments of the narration of awe using explicit definitional criteria, though additional validation is needed. This work contributes to the empirical basis for direct observation coding systems to help us better understand the expression of awe and train computational methods to automatically identify and characterize these moments.

## Figures and Tables

**Table 1:** Definitions and examples of vastness, cognitive disruption, accommodation and ineffability expressions.

Primary Features	Definition	Sample Linguistic Markers	Illustrative Example
**Vastness**	Expressions relating to anything being much larger than the self (i.e., physically, socially, conceptually).	Lexical items referring to size or relative size of the physical world or iconically large things (e.g., NYC, Redwood Trees, Ocean), similes or metaphors tied to expansive concepts (e.g., time, space, infinity, the universe).	“I was in that space where there was this, in front of me, like this cascade, and it was a cascade of stories, it was the infinite possibilities of all the stories that could ever exist.” (Participant E, Integration.)
**Cognitive Disruption**	Discussions related to an experience that is radically new or outside the current cognitive framings of the participant.	Statements of inherent contradiction or veridical paradox, superlative or extreme evaluative predicates (e.g., *amazing, devastating*).	“It really opened up a completely different dimension of the experience where I was in that space of non-judgement and infinite possibilities. Everything and nothing and everything and nothing and everything and nothing. It didn’t matter. And that was the most amazing thing. It didn’t matter. It was not good or bad, it just didn’t matter, didn’t matter, didn’t matter. So that was, that was, that was, yeah, really, really amazing.” (Participant E, Dosing.)
**Accommodation**	Expressions suggesting that mental structures have shifted or expanded to make sense of this new experience/cognitive disruption; reflecting meaning or sense-making (N/A without cognitive disruption).	Stative verbs (*guess/think*) or factive verbs (*know/realize*) with clausal complements; explicit mention of “meaning” or “understanding”.	“That one’s a little—it’s funny because that one, like I said, was a crystal clear message in the session, but I don’t exactly know what it means. I’m still trying to figure out what about that—the ashamed part—and how that applies to me.” (Participant K, Integration.)
**Ineffability**	When a participant is unable to articulate some aspect of their experience because of its profound nature and due to the strength of their affective response.	Long pauses (+2 s), word- and sentence-level restarts, dedicated discourse markers (i.e., ineffability *just*), explicit acknowledgement of expressive difficulty.	“I don’t know how- it’s hard to put into words…” (Participant I, Dosing.)

## Data Availability

The datasets presented in this article are not readily available because they represent protected health information. Requests to access the datasets should be directed to the corresponding author.
